# On the Action of Diuretic Medicines

**Published:** 1838-01-01

**Authors:** 


### On the Action of Diuretic Medicines. By *Dr. C. G. Mitcherlich.*

(From the Archiv. Für Anatomie, Physiologie, von Dr. J. Müller, Jahrgang 1837. Heft 3.)

We trust the following copious analysis of an essay coming from so distinguished an authority as Dr. Mitcherlich, on so interesting and important a subject, will not be considered out of place in a journal devoted, as ours ever has been, to the advancement and improvement of PRACTICAL MEDICINE. If there be any one class of therapeutic agents more abused than another, in the hands of the mere routine practitioner, it is the class of diuretics. In treating the disease for which diuretic medicines are principally employed, namely dropsy, it has been and still is too much the practice to prescribe for a mere name, the pathological condition on which dropsy may depend, and the primary disease of which the dropsical effusion is but a symptom, being entirely left out of consideration. Did the physician, when prescribing diuretics, recollect that most of those articles of the materia medica which are specifically and κατ' ἐξοχὴν classed under this head, are powerful local stimulants, and that it is by their revellent effects combined with the diuresis which they occasion, that they produce beneficial results, we should not so often see them prescribed in cases wherein the primary affection is seated in the kidneys, this affection being accompanied with inflammation or at least irritation of those organs, and consequent diminution of the urinary secretion; in which from an exclusive attention to the latter circumstance their use appears to the superficial observer to be indicated. In such cases their employment cannot fail to add to the mischief, the best diuretics obviously being the lancet and the antiphlogistic plan of treatment, which, by removing the cause of the diminished secretion, would tend indirectly to establish its perfect restoration. For a knowledge of the fact that dropsical effusion is frequently dependent on disease of the kidneys, and that the mark of this dependance is plainly afforded by the albuminous state of the urine, we are indebted to the pathological researches of Drs. Bright, Christison, Gregory and Osborne. We shall without further preamble present the Essay to our readers. The urinary secretion removes superfluous water and other materials, which are no longer of use in the animal economy, from the mass of blood, and, in connexion with those organs destined to similar functions, keeps up the healthy composition of the blood.

Should the urinary secretion be stopped, serious diseases are occasioned. The extirpation of both kidneys, according to the experiments of Prevost and Dumas, produces death before the tenth day, and the blood is then found to be thin, more fluid than natural, abounding in urea; serum also is effused into the cerebral cavities, the mucous membrane of the lungs is covered with mucus, and a quantity of thin fæces with a considerable quantity of bile is found in the intestinal canal. Should the suppression of the urinary secretion not take place suddenly, as in the experiments on animals just mentioned, the disease (ischuria renalis) in such case proceeds as follows: a urinous taste is produced in the mouth, the cutaneous, salivary and intestinal secretions are increased, and emit a urinous smell; violent fever, and dyspnœa ensue, severe head-ache with dizziness, somnolency, delirium, convulsions, and apoplexy or suffocation, terminate life. On instituting a post-mortem examination, there is then found a copious collection of water in the different cavities, and the odour of urine is also distinctly perceived. The non-secretion of the urine from the blood makes this latter fluid thin, produces increased secretion from the other secreting organs, is followed by effusion of serum into the cavities, and gives rise moreover to peculiar symptoms, which in all probability depend on the action of the constituents of the urine. Should the urinary secretion be gradually diminished, without becoming entirely suppressed, the results are of a different kind, and dropsy becomes established; this for instance takes place in degenerescence of the kidneys. Cases are on record in which no discharge of urine took place for months or even for years. Richardson mentions a case in which no urine was ever passed, and that without any inconvenience. Such observations however are to be considered extraordinary phenomena, and stand in need of confirmation.

The urine in a healthy individual has an acid re-action, a specific gravity of 1,005—1,03, at most of 1,025 (Prout), and should contain from 4 to 8 of solid constituents. Great varieties are found here, according as the urine of the morning or the evening be made the subject of examination, according as the condition be after copious drinking, or a long thirst, &c. The quantity of the urine evacuated daily by a healthy man varies considerably, and depends on the quantity of the fluids secreted by the skin, intestinal canal, &c. and on the quantity of the fluid and solid food consumed by the individual.

The ordinary constituents of the urine are: free uric acid, free lactic acid, lactate of ammonia, sulphate of potass and of soda, phosphate of soda, biphosphate of ammonia, chloride of sodium, muriate of ammonia, fluoride of calcium, phosphate of lime and magnesia, silica and urea, as also an indeterminate quantity of animal matter. Besides this, the urine also contains some mucus from the bladder.

These constituents are constantly found in the urine of an individual in the state of health, but not always in the same quantity, nor in the same relative proportions. The urine of children is poorer in urea and uric acid than that of adults. The cutaneous transpiration exercises very considerable influence on the urinary secretion, and the greater the quantity of fluid produced from the body by that means is, the more concentrated is the urine, and in the less quantity is it secreted. Accordingly we find a great difference in warm seasons and in Winter with respect to the secretion of urine, as also in a moist and dry atmosphere. When the intestinal discharges are copious, a smaller quantity of urine is secreted. Nor can we, under such circumstances, raise the urinary secretion by means of medicine, as in the opposite case. Drink exercises still greater influence, as the urine flows so much the more abundantly, and becomes poorer in solid constituents, the more we drink. Water is carried away by the skin, &c. but in considerable quantities also by the kidneys. On this account the urine is more concentrated in the morning than in the evening, because less is drunk at night. Should the drink contain other materials in solution, these also frequently change the composition of the urine, as they are partly excreted through the kidneys, whilst some of them influence the formation of other materials.

The food produces an important change in the urine, as many substances which are absorbed after digestion, are not converted into materials of nutrition, and are eliminated with the urine ; they may have suffered a change during their circulation or not. Thus after digestion the urine is rich in urea, uric acid and salts.

The urine undergoes very important changes in disease both with respect to its quantity, and also with respect to its composition. These differences we have here noticed but very briefly. The quantity of the urine is very much increased in diabetes, at the same time that a great quantity of water is drunk. The urine diminishes very much and contains much solid constituents, if violent sweating or diarrhœa carry off a considerable quantity of fluids from the body, and if, as in dropsy, a great quantity of fluid be deposited into the cellular tissue or into the cavities of the body. The composition of the urine also varies very considerably in diseases, on which subject however, but few investigations have been made. Thus for instance, in paralysis proceeding from the brain or spinal cord, we frequently find urine with an alkaline reaction ; in diabetes mellitus it is found to contain sugar ; in dropsy it often contains albumen ; in gout and rheumatism uric acid and urate of ammonia in great quantity.

Most articles of the materia medica also change the urinary secretion in a very high degree, as well, with respect to quantity, as to its composition. They produce these changes partly by acting on other organs as it were antagonistically, as purgatives for example, partly by a direct action on the kidneys, as saline medicines, and partly by producing a change in the blood. Some diminish the urinary secretion, as purgatives, opium, &c., others increase it, and thence are called diuretics. The changes in the composition have been scarcely at all investigated, and we only know that many medicinal substances are again found in the urine, (salts, colouring matter, &c.) and that others change its odour (turpentine, asparagus, &c.)

Those medicinal substances which increase the quantity of the urine, yield thereby a urine which is deficient in solid constituents, and which accordingly has a less specific gravity than before. This is frequently anticipated, in as much as such urine generally continues clear on cooling, tho' this opinion is not confirmed by experiment. In diabetes the urine is often very clear, and yet not unfrequently it has a very great specific gravity. Accordingly the conclusion is by no means correct in every instance, that a clear urine is less concentrated, than a urine which, on cooling, yields a precipitate. The uric acid, for instance, which is very scantily soluble in water, falls to the bottom on the cooling of the urine, as soon as it is secreted from the blood in greater quantity, without the urine still being of a great specific gravity.

For the investigation of these facts I have collected some observations made on dropsical patients, and though several salts were present, I constantly found *that the specific gravity of the urine is diminished, if its quantity be increased.* In one case the urine was of the specific gravity 1,022, and on the following day, after 15 grains of carbonate of potass in solution were taken every three hours, its specific gravity fell to 1,01. The quantity of the urine secreted was at this time increased to about double. In the other cases, this difference, though less palpable, was still evident. We possess similar results with respect to other secretions : thus in the case of salivation by mercury, the specific gravtiy of the saliva is diminished to

1,0021—1,0038 (instead of 1,0062—1,0088). It is probable that every secretion, which becomes more copiously secreted than before, in consequence of the employment of a medicinal substance, also becomes poorer in solid constituents than before. The secretions of the intestinal mucous membrane are always rendered very watery after large doses of purgatives. The differences which the individual articles present, have not as yet been investigated. This increased secretion of urine with a small proportion of solid constituents by means of diuretics, and consequently of less specific gravity than the blood, is a fact, which, as I shall presently shew, is of considerable importance. Investigations on the changes in the relative quantity of the constituents of the urine by diuretics we do not possess. In salivation after the use of mercury the relative quantity of the constituents of the saliva is essentially changed; the quantity of the salts has increased, while that of the salivary material has become less. On the appearance of new substances in the urine after the use of diuretics we possess no facts, only some few conjectures; thus, during the use of turpentine, we find the urine of a peculiar odour. Those medicinal substances, which are easily found in small quantity in solution with organic substances, are also found again in the urine, as the salts for instance. The other substances are not detected in the urine.

Diuretic medicines are very various. We have medicines, which in a healthy individual produce an increased urinary secretion by increasing the action of the kidneys, (diuretics properly so called). Other medicinal substances, on the contrary, produce their diuretic effect by removing the cause of the diminished urinary secretion, (diuretics in a therapeutical sense); the increased secretion in this case is only an adventitious result. Water itself increases the secretion of urine, as it must be constantly removed again through the skin, lungs, intestinal canal, and kindeys.

### I. DIURETICS WHOSE PHYSIOLOGICAL ACTION IS TO PRODUCE AN INCREASED SECRETION OF URINE.

These occasion a more copious secretion of urine than existed previously, if there be a sufficient quantity of fluid in the body. If the kidneys be the seat of irritation or inflammation, these increase it, and inflammation is even occasioned by violent diuretics.

To these belong:—

1. *Acrid Diuretics*—which act directly on the kidneys. Inflammation of the kidneys is increased by them. We observe, in many instances, strangury and the secretion of bloody urine produced, in some cases, inflammation of the bladder and of the kidneys, if these medicines be persevered in and given in large doses. Those substances produce inflammation externally only in those parts to which they are directly applied, but nowhere else. To these belong cantharides, squill root, colchicum seeds and root, mustard seeds, Mezereon bark, &c.

2. *Stimulating Diuretics.* These excite all the functions more or less. The acceleration of the circulation is here of great influence, because in a given time a greater quantity of blood is sent to the kidneys than before. But stimulating medicines act directly also on the kidneys, because small doses of them, which are followed by an almost imperceptible acceleration of the circulation, manifestly increase an inflammation of the kidneys. This excitement is the more violent, the more closely the exciting substances approximate to acrid substances, as for instance, turpentine. To this class belong alcohol, ether, the stimulating ethereal oils and resins, balsams, &c., and consequently all medicinal substances which contain these ingredients as active principles.

3. *Saline and Alkaline Diuretics.* These substances act at one time by changing the mass of blood, as I shall shew presently, and secondly also by directly exciting the kidneys. No inflammation is excited by these, but one already existing is exasperated by them. They act here in the same way as they act on the first place of contact (the stomach, wounds, &c.) where they increase the secretion, without producing similar phenomena in the course of the circulation, whilst at the same time they produce even an antiphlogistic effect. To these belong potass and soda, and their combinations with strong and weak acids, &c.

These diuretic substances act directly on the kidneys, after being absorbed and taken into the mass of blood. The proofs are as follow:

A. The neutral salts and alkaline substances we can find again in the urine. The acid is frequently changed, but the base continues. After the use of alkaline substances the urine very soon becomes alkaline. We find that a given quantity of urine contains much more of these salts than an equal quantity of blood. The acrid and stimulating substances have not yet been detected in the urine by chemical examination, for the peculiar odour of the urine occasioned by the use of oil of turpentine is no proof of the presence of the latter, but merely points out a change in the urine.

B. The degree of local action bears no proportion to the increase of the diuresis. After large doses of acrid medicines vomiting and purging take place, and even inflammation may follow: the local action is accordingly very considerable, but the medicinal substance is soon evacuated again, and therefore cannot be absorbed; consequently either no increased diuresis follows, or at least a diuresis very slightly increased. If, on the contrary, the substance be administered in doses so regulated, that its local action is not too violent, and that it continues for a sufficient length of time in contact with the intestinal mucous membrane, so as to become absorbed, it then acts most violently on the kidneys. The same may be said of stimulating, saline, and alkaline diuretics, which act most powerfully, when the local action is not too great. Accordingly we cannot explain the diuretic action sympathetically through local irritation of the intestinal canal.

C. The time after which the increased diuresis commences corresponds with the time in which absorption can follow. All sympathetic phenomena are observed instantaneously; and hence, if these substances acted in this way, the increased diuresis should also take place immediately. But this does not follow till a later period; often not till after the lapse of several hours, in which time absorption may take place.

D. The same series of changes which these substances produce in the intestinal canal on wounds, &c., we also find produced in the kidneys, if they be given in a sufficient, but not in too large a quantity. Cantharides and produce inflammation on the epidermis in wounds, in the stomach, &c., and by the continued administration of large doses of this substance, inflammation of the bladder and kidneys also takes place. The stimulating diuretic substances increase inflammation in the stomach, &c., and exasperate inflammation in the kidneys. The salts which can be detected in the urine by chemical examination act in a manner so as to increase the inflammation in the first place of contact, and after absorption in the kidneys, so as to diminish, on the contrary, the inflammation, which makes its appearance in other organs.

The principle on which these substances increase the urinary secretion, and the manner in which this effect is brought about, we know not. We only know that the salts are secreted with the urine in a more concentrated form than they exist in the blood, and that the direct action of the other substances on the kidneys can be shown with the greatest probability. This is what is called a specific action.

In morbid deposition of serum in the meshes of the cellular tissue, or in the different cavities of the body, as in dropsy, absorption of the serum, and the cure of the disease, frequently take place simultaneously with increased diuresis after the employment of these diuretic substances. The diseases which depend on an acrimony of the blood are often relieved and cured by these substances; to this class of diseases may be referred cutaneous eruptions, &c.

The cure of dropsy by these substances has been generally accounted for in such a way, as that a specific action on the lymphatic vessels has been assigned to them, and this action was derived from a power inherent in them of promoting absorption. No substance has been proved to possess such a specific action on these vessels; but the increase of absorption is probably owing to a change effected in the blood.

According to our present knowledge of absorption, and considering all the phenomena presented by the increased diuresis through the medium of the substances now mentioned, the absorption of the serum in dropsies, and the cure of these diseases may be explained in the following manner. The first momentum is the stimulation of the kidneys, and the secretion of a urine deficient in solid constituents. If we now compare the composition and specific gravity of this urine with those of the blood, the blood must necessarily become richer in solid constituents, inasmuch as it possesses much more of these ingredients than the urine secreted. But in consequence of this, the blood has a greater affinity for fluids than before, just as we see that violent thirst is occasioned if much water be abstracted from the body by excessive cutaneous perspiration. In the same manner absorption is caused here by the increased attraction of the blood for serum. A similar physical phenomenon is observed, if a glass tube, open at both ends, be closed at one end with a membrane, and be half-filled with a concentrated solution of a salt, and be immersed to this depth in pure water, the concentrated solution attracts the water through the membrane, and rises in the glass tube.

For the cure of those diseases by means of diuretics, which are said to depend on acrimony, some have endeavoured to account by ascribing to these medicines a power of producing a change in the blood. But this is not proved in such a way as to enable us to explain any thing with certainty. It is much more probable that these acrimonious admixtures with the blood, if they really are the occasion of these diseases, are removed along with the urine, in consequence of the action of the kidneys being called forth.

Analogy is also in favour of this explanation, as these same diseases are cured by cathartics, which produce an irritation of the intestinal canal, and thereby an increased secretion of its mucous membrane. In the same manner as diuretics increase the secretion of the kidneys, discharges of fluid take place in the latter case. Purgatives act neither sympathetically, nor directly on the lymphatic vessels, but by exciting the secretion of the intestinal canal, and separating a watery fluid from the blood.

### II. Diuretics whose Therapeutic Action occasions an Increased Secretion of Urine.

The diuretic means which call forth an increased secretion of urine in disease are very various. Every means which can remove the cause of a diminished urinary secretion belongs to this head.

The abstraction of blood by venæsection, leeches, &c., bring about a more copious secretion of urine, if an inflammation of the kidneys be present, if inflammation of other parts, or an inflammatory fever be followed by a scanty secretion of urine. By this means the inflammation, which was the cause of the diminished secretion of urine, is put a stop to.

Neutral salts, and alkalies also, produce a more copious secretion of urine. In the case of inflammation, the cause of the diminished diuresis is checked by these means, if the inflammation has not its seat in the intestinal canal and kidneys. In impeded circulation occasioned by intumescence, or other morbid states of the liver, spleen, and other organs, dropsy and a diminished secretion of the urine occur. Many of these causes of impeded circulation are curable by the means just mentioned, and with the removal of the cause the serum is again taken up by the vessels. They here act as resolvents.

Temperant remedies (acida vegetabilia) act diuretically in inflammations, by their mitigating the symptoms of inflammation.

Emollients obviously occasion an increased diuresis in inflammation of the kidneys. They mitigate the inflammation, and thereby increase the secretion of urine.

Tonics also increase diuresis. In consequence of an atony of the solids, which atony coincides more or less with a blood poor in solid constituents, dropsy takes place. This dropsy is removed by the digestive function being strengthened so as to form more blood, and blood of a good quality (by bitters, bark, iron, &c.), and so as to increase the tone of the tissues (by iron, bark, and other astringents). By these means the cause of the dropsy, viz. the defective formation of blood and the atony of the tissues, is removed.

Digitalis possesses the peculiar property of lessening the heart's action, of bringing down the pulse from 80 to 60 or 50, &c., and of diminishing the strength of the pulse, whereby it sets the kidneys to work simultaneously as an active remedy. Accordingly, if the dropsy is a consequence of an enlargement or hypertrophy of the left ventricle, digitalis mitigates the violent action of the heart thereby produced, and the dropsy disappears for some time; but this does not cure the heart disease, and the dropsy accordingly returns. By lessening the heart's action, retarding the circulation, and by directly increasing the urinary secretion, digitalis is also useful in inflammatory dropsy, more especially if exudation impends or has already occurred, and the inflammation be first broken down by venæsection, &c.

The acrid (scharfen) medicines are diuretic, if the diminished urinary secretion is a consequence of torpor of the kidneys. Whether it results from paralysis depending on the brain and spinal cord, can only be conjectured, but not known with certainty.

Stimulating remedies increase the diminished urinary secretion in the same case, and especially in deficient activity of the circulation. The dropsy is seldom cured by these means alone, as in torpor of the kidneys acrid remedies are to be preferred; and dropsy very seldom occurs in consequence of the heart's inaction, without organic disease. But these remedies are of the utmost importance when the morbid matter exists in the blood, keeps up the disease, and is not eliminated. They accelerate the circulation, direct more blood to the kidneys in a given time, to the skin also, &c., and call into action at the same time the functions of those organs. Accordingly, copious secretions often follow the employment of these means, crises take place, and stimulants may therefore in such cases become tonic remedies, &c., by removing the cause which interferes with the action of the kidneys.

Antispasmodics are just as various as the causes of spasm. Almost all medicines may act as antispasmodics under certain circumstances, but some pre-eminently so, in primary spasmodic diseases of an entirely different kind, as narcotics, for instance, and those with which we are able to produce a powerful counter-irritation. If the diminished urinary secretion be a consequence of spasm, the antispasmodic medicine is, in a therapeutical point of view, a diuretic. Thus we often see an increased diuresis occur in consequence of an emetic, and that either in consequence of the general counter-irritation, or of the general shock and excitement given to the system by this means. In this way opium, which in healthy persons diminishes the urinary secretion, may, by removing spasm, call forth a more copious secretion of urine.

If from this point of view we consider the action of those remedies which are followed by an increased urinary secretion in dropsy, we must next turn our attention to the primary affection, which is followed by dropsy as a symptom in the progress of the disease. If we connect both these series of facts with our present experience at the sick bed, we may then consider the following points as established.

The dropsy which is a consequence of a so-called inactivity of kidneys, or of a diminished secretion from the skin, &c., is diminished or cured by those remedies which directly excite the action of the kidneys. In the first case, the cause of the disease is removed; in the latter, the increased activity in the kidneys takes on the function of the skin, &c., and there then remains for us the problem of regulating the function of the skin, which is more easily accomplished, if the accumulation of water is first stopped.

The dropsy, which is a consequence of a structural change in the kidneys, is but seldom cured. The diagnosis is frequently uncertain, and, above all, the kind of structural change for the most part continues unknown to us till death. Medullary sarcoma and similar organic diseases of the kidneys are at present still incurable. The structural change, which Bright, and subsequently Gregory, Christison and Osborne, have detected in the kidneys, appears to be increased by acrid, stimulating and saline diuretics, and to require partly the abstraction of blood, partly purgatives, as well as careful attention to the cutaneous transpiration.

The dropsy which is a consequence of an impeded circulation, is cured by removing the cause of the latter. A tumor (Balggeschwulst), &c., may create œdema by pressure on the veins, and in such a case the œdema disappears with the removal of the tumor. Tumors of the liver, spleen, &c., depositions in these organs, and degenerations of them, impede the return of the blood, and in this way may occasion dropsy. If these depositions and swellings be still resolvable, the dropsy may be removed by neutral salts and alkaline remedies, which act on these organs by changing the mass of blood. What are called obstructions in the liver, spleen, in the portal system, and in the lymphatic vessels, are often removed by these resolvents, often cured by promoting the secretions of the liver and capillary vessels of the intestinal mucous membrane (cathartics in small and large doses). But incurable degenerations often occasion the dropsy by impeding the return of the blood, and in such a case no remedy cures the dropsy.

The dropsy which is the consequence of inflammation, as hydrothorax after pleuritis, is removed by those means which mitigate or remove inflammation, whether by a change in the composition of the blood, or by retarding the circulation, and which have a solvent action on the effusion, if it is no longer fluid, and at the same time effect an increased diuresis. To these belong bloodletting, the neutral salts, and alkaline remedies, which diminish inflammation by changing the blood, and at the same time act on the exsudation. Mascagni showed that carbonate of potass dissolves the solid exsudation in pleuritis, and accordingly this remedy is employed with the best results when auscultation and percussion have detected an effusion into the thoracic cavity. To this class also belongs digitalis, in as far as it retards the circulation and excites the action of the kidneys.

The dropsy which is a consequence of atony depends on a deficient formation of blood, and insufficient contractility of the tissues, which are not properly nourished. In such a case the digestive powers are to be strengthened by bitters, quinine, iron, &c., and from mere bitters the change may be made to astringent remedies. Herewith the removal of the cause, the dropsy, which is but a symptom, disappears.

The dropsy which is a consequence of dilatation and hypertrophy of the left ventricle, is often removed for a considerable time, but again returns. By bloodletting, should plethora exist at the same time, a temporary improvement is effected, and by the use of digitalis, which diminishes the abnormal energy of the heart's action, the dropsy, if it have not gone too far, is often removed for some time, but returns, if the effect of the remedy has ceased for any length of time. The cause of the dropsy is in this case suspended for a long time by diminishing the heart's action, but the hypertrophy is not cured thereby.

If the cause of the dropsy, or rather the primary disease, is not to be detected, several modes of treatment are employed, according to the view which the physician adopts in determining the casual relation.

If the case be one of hydrops saccatus, those remedies are seldom of use, and a rational line of treatment verified by experience has not been yet established.

If the effusion (as, for instance, into the pleura) be no longer fluid, and the serous membrane be at the same time very much affected, these means are sometimes of use after paracentesis, but seldom effect a perfect cure. Saline and alkaline remedies hitherto proved most serviceable in these cases, and that in the two-fold relation, as diuretics in the more limited sense of the word, and as resolvents.

